# Progress in Research on the Mechanisms Underlying Chloroplast-Involved Heat Tolerance in Plants

**DOI:** 10.3390/genes12091343

**Published:** 2021-08-28

**Authors:** Chu Zeng, Ting Jia, Tongyu Gu, Jinling Su, Xueyun Hu

**Affiliations:** 1 College of Bioscience and Biotechnology, Yangzhou University, Yangzhou 225009, China; 172101127@yzu.edu.cn (C.Z.); mz120191171@yzu.edu.cn (T.G.); 182102213@yzu.edu.cn (J.S.); 2 Joint International Research Laboratory of Agriculture and Agri-Product Safety of the Ministry of Education of China, Yangzhou University, Yangzhou 225009, China; tingj2012@yzu.edu.cn; 3 Key Laboratory of Plant Functional Genomics of the Ministry of Education, Yangzhou University, Yangzhou 225009, China

**Keywords:** chloroplasts, heat stress, chloroplast retrograde signal, heat tolerance

## Abstract

Global warming is a serious challenge plant production has to face. Heat stress not only affects plant growth and development but also reduces crop yield and quality. Studying the response mechanisms of plants to heat stress will help humans use these mechanisms to improve the heat tolerance of plants, thereby reducing the harm of global warming to plant production. Research on plant heat tolerance has gradually become a hotspot in plant molecular biology research in recent years. In view of the special role of chloroplasts in the response to heat stress in plants, this review is focusing on three perspectives related to chloroplasts and their function in the response of heat stress in plants: the role of chloroplasts in sensing high temperatures, the transmission of heat signals, and the improvement of heat tolerance in plants. We also present our views on the future direction of research on chloroplast related heat tolerance in plants.

## 1. Introduction

Global warming and frequent extreme weather events have brought severe challenges to plant production. By the end of the 21st century, the global temperature is expected to increase 2.0–4.9 °C, with a median of 3.2 °C and a 5% (1%) chance that it will be less than 2 °C (1.5 °C) [1]. Temperature is one of the major environmental factors that affects plant growth and development. Temperatures persistently above what is optimal for plant growth and development may cause heat stress [2]. Heat stress has negative effects on plant photosynthesis, respiration, root growth and development, pollen viability, seed filling, and so on [3]. Therefore, heat stress will not only lead to reduced agricultural production and threaten food security but also reduce the productivity of plants and destroy the existing ecological balance. For example, it is predicted that for every 1 °C increase in the global temperature, the yield of wheat will decrease by approximately 6% [4]. Therefore, in recent years, the effect of high temperature on plants and the mechanisms underlying their high-temperature tolerance have gradually received more attention from plant scientists. The mechanism of plants’ response to heat stress is very complicated. Briefly, heat stress may alter plasma membrane fluidity, which may activate Ca^2+^ channels, resulting in an influx of Ca^2+^ ions and activation of the Ca^2+^ signalling pathway. In addition, heat stress affects protein stability and promotes ROS over-production, leading to protein misfolding and metabolic imbalance. All these alterations in plants caused by heat stress will alter the expression of heat stress responsive genes, such as *HSFs* and *HSPs*, leading to plant acclimation to heat stress, if the heat stress is non-lethal to plants [5]. Recently, it was discovered that small RNAs and epigenetic and chromatin-based modifications are also involved in plant heat stress response and memory [5,6].

As the site of plant photosynthesis, chloroplasts are one of the organelles most sensitive to high-temperature stress. Researchers have made much progress in understanding the response of chloroplasts to high-temperature stress, the transmission of heat signals by chloroplast retrograde signalling, the mechanism by which nuclear-encoded genes regulate chloroplast heat resistance, and their application to improving the heat resistance of plants. This paper reviews the research progress in this field from these aspects and discusses the existing problems. Finally, the prospect of improving plant heat tolerance through the regulation of chloroplast protein expression is discussed.

## 2. Chloroplasts Are Organelles That Are Sensitive to Heat Stress

Heat stress can affect the photosynthesis of plants and can cause an imbalance in their intracellular energy status. It was reported that moderate heat stress (e.g., 35–40 °C) can cause chloroplast enlargement and increase the number of plastid vesicles in chloroplasts, suggesting that it significantly changes the structure of chloroplasts and thylakoids [7]. This is mainly because the high temperature destroys the thylakoid membrane by changing the redox state in the chloroplast. Therefore, the photochemical reaction in the thylakoid lumen and the carbon metabolism in the stroma are both severely impaired [8,9,10,11]. In addition to the direct damage to PSI, PSII, cytochrome b6f, and Rubisco, heat stress can also block different redox reactions and various metabolic reactions [11,12].

The photosystem is easily destroyed by heat stress. PSII is thought to be the most sensitive part of the chloroplast to heat stress and is the primary target of heat stress. Under heat stress, the integrity of PSII is damaged, so photosynthetic electron transport is affected [12,13,14]. Heat stress causes the dissociation of the oxygen-evolving complex from PSII, which in turn affects electron transport on the acceptor side of PSII [15]. In addition, oxidative stress occurs as a consequence of heat stress. In the process of photosynthesis, active singlet oxygen is generated, which destroys the reaction centre protein D1/D2 of PSII, thereby promoting the repair cycle of PSII [16]. Furthermore, sustained heat stress inhibits PSII repair by inhibiting the synthesis of PSII proteins, resulting in a decrease in the photosynthetic efficiency of plants [17]. In cyanobacteria, a certain amount of ROS that is produced under moderate heat stress conditions might not cause severe damage to PSII but can prevent PSII repair [18]. The structure, composition, and function of PSI also undergo significant changes under heat stress [19]. When the temperature gradually increases, the antenna protein of PSI gradually dissociates from the core protein of PSI under heat stress; that is, the PSI–LHCI supercomplex disintegrates, and then the phenomenon of LHCI aggregation arises [20,21,22].

The protein translation system of chloroplast is another site that can be easily damaged by heat stress. As a semiautonomous organelle, chloroplast has their own genome and gene transcription and translation system. The chloroplast genome encodes less than 10% of chloroplast proteins that are the major components of multiple photosynthetic systems. Heat stress hinders PSII repair, mainly by destroying the protein translation system in chloroplast. EF-Tu is a protein conserved in prokaryotes and in eukaryotic mitochondria and chloroplasts. EF-Tu in *Arabidopsis* chloroplasts, named with Rabe1b, rapidly accumulates and becomes insoluble under high-temperature stress, leading to a reduction in its activity [23,24,25]. This protein has low heat tolerance. Additionally, chloroplast RPS1, a chloroplast ribosomal protein, is also a heat-responsive protein. It responds to heat stress at protein level, but not at transcriptional level [26]. In *rabe1b* and *rps1* mutants, the expression of nuclear heat-responsive genes regulated by the HsfA2 is significantly inhibited [26,27]. Besides translation, the transcription of chloroplast-encoded genes is also altered by heat stress [28].

Chlorophyll metabolism may become imbalanced when plants are under stress conditions. Chlorophyll is the main photosynthetic pigment in the photosynthetic system and is the main molecule responsible for absorbing light energy and transferring electrons in the process of photosynthesis. Under non-stress conditions, the rate of chlorophyll synthesis and degradation in leaves is relatively steady [29]. The yellowing symptoms of leaf senescence or chlorosis in plants under heat stress are all caused by the decrease in chlorophyll content [30,31]. At present, the mechanism underlying the reduction in chlorophyll content caused by heat stress remains unclear. Studies on bentgrass showed that heat stress increased the gene transcription level and activity of PPH, a key enzyme in chlorophyll degradation, but did not significantly change the transcription of chlorophyll synthesis genes or the activity of key enzymes [31]. Due to the damage to PSII caused by heat stress, part of the chlorophyll will also undergo turnover during PSII damage and repair. That is, under the action of CHLG and CLD1, some chlorophyll can be reused [32,33]. Actually, the transcription of *CHLG* and *CLD1* is induced by heat stress, and plants lacking CHLG or CLD1 are sensitive to heat stress, indicating that chlorophyll turnover is an important part of plant heat responsiveness.

Moreover, the dark reaction of photosynthesis is inhibited by heat stress too. Rubisco is not only a carboxylase necessary for the fixation of CO_2_ in photosynthesis but also an indispensable oxygenase in photorespiration. Rubisco catalyses the carboxylation of the five-carbon sugar ribulose-1,5-bisphosphate in the Calvin cycle. However, the catalytic activity of Rubisco is relatively low and requires the participation of RCA [34]. Under moderate heat stress, the activity of RCA is inhibited, resulting in a significant decrease in the activity of Rubisco [34,35,36,37]. Therefore, RCA is considered to be the limiting factor of photosynthesis at high temperatures [10]. At the same time, a high temperature will reduce Rubisco’s affinity for CO_2_, making it easier for Rubisco to catalyse the oxygen addition reaction in photorespiration. Besides inhibiting photosynthesis, heat stress also enhances photorespiration, resulting in the hindrance of plant growth and development [11].

## 3. Chloroplast Retrograde Signalling Regulates the Response of Plants to Heat Stress

The mechanisms of plant heat perception are relatively poorly understood, although heat perception is the first step of plant heat sensing and its primary signal transduction. It has established that phytochrome B, a light-sensing protein, is one of the temperature sensors. In addition, other photoreceptors that respond to heat stress may be also involved in plant heat sensing [38]. Since photosynthetic components are some of the main targets of heat stress, chloroplasts are considered sensors of thermal stress. Recently, it was uncovered that chloroplasts have the ability to sense absolute high temperature [39]. High temperature promotes an increase in Ca^2+^ concentration in the chloroplast stroma and does not change the Ca^2+^ concentration in the cytoplasm. This phenomenon suggests that the thermoreceptors of plants may be located in chloroplasts. The temperature sensor is regulated by a Ca^2+^-sensing protein (CAS), located on the thylakoid.

When chloroplasts experience heat stress, they subsequently transmit chloroplast retrograde signals to the nucleus, thereby inducing the expression of heat-responsive nuclear genes, especially the genes coding HSPs.

ROS-dependent chloroplast retrograde signalling is considered an early response of plants in the adaptation to environmental stress [40]. Among them, singlet oxygen signalling is the most intensively studied pathway. Stress leads to the accumulation of singlet oxygen, which in turn leads the plant to respond to the stress, including by changing the expression of many nuclear genes and increasing the synthesis of stress-related hormones such as salicylic acid, ethylene, and jasmonic acid [41]. However, the half-life of singlet oxygen is very short, and it is unlikely to escape from the chloroplast and enter the nucleus as a chloroplast retrograde signal. Therefore, the more stable second messenger molecules triggered by singlet oxygen may be those that activate the signalling pathways that control nuclear gene expression [42]. 

Because β-carotene can be oxidized to β-cyclocitral by singlet oxygen, it was discovered that β-cyclocitral participates in singlet oxygen-dependent chloroplast retrograde signalling [43]. As a stress signal, β-cyclocitral can reprogram gene expression, shifting plant cells from active growth to cellular defense toward stress. H_2_O_2_ is the most stable ROS. In the early stage of heat stress, H_2_O_2_ is a major signal activating HSFs, such as HSFA1a and HSFA1b in *Arabidopsis* [44]. H_2_O_2_ is believed to be able to penetrate the chloroplast membrane through free diffusion, thereby triggering mitogen-activated protein kinase-mediated signals in the cytoplasm [45]. Preventing the accumulation of H_2_O_2_ can inhibit the expression of heat-induced genes, indicating that H_2_O_2_ is an essential component of the heat signalling pathway. H_2_O_2_ in chloroplasts also plays an important role in the regulation of heat stress memory in plants [46]. Although many studies suggest that H_2_O_2_ is a signalling molecule, there is still a lack of clear evidence of its role as such [11]. In addition, whether H_2_O_2_ has different roles in different organelles and whether it can diffuse freely in plant cells remains unclear.

Some other metabolic molecules were reported that function in chloroplast retrograde signalling pathways that regulate plant heat responsiveness. In *Chlamydomonas reinhardtii*, tetrapyrrole Mg-Proto IX and hemin are considered the key molecules in the regulation of nuclear gene expression by chloroplast retrograde signalling. Approximately 982 genes can be regulated by these tetrapyrrole molecules, of which 51% are also activated by heat stress [47]. Vitamin E is involved in the high-temperature adaptability of plants. It regulates the accumulation of the chloroplast retrograde signalling molecule PAP, a retrograde inhibitor of the nuclear exoribonucleases. Subsequently, PAP positively regulates the production of intracellular microRNAs, especially miR398, thereby improving the heat tolerance of plants [48].

Furthermore, several chloroplast proteins that have a function in regulating chloroplast retrograde signalling pathway are also involved in transmitting heat stress signalling. The chloroplast retrograde signalling pathway regulated by GUN5, the H-subunit of Mg-chelatase, activates the nuclear gene *HSP21* in response to heat stress. HSP21 enters the chloroplast to protect the core subunits of PSII, D1, and D2, making D1 and D2 more heat resistant [49]. The chloroplast translation system is a key system that generates retrograde signals to activate the heat-responsive expression of *HsfA2* and its target genes [26]. CIA2 is a transcription factor that is localized in both chloroplasts and the nucleus. It is involved in the regulation of the response of *Arabidopsis* to ultraviolet-AB radiation, high light, and heat shock, and it is critical for the regulation of ribosome assembly and maturation [50]. It may be involved in the transmission of retrograde signals by chloroplasts in response to stress; however, the detailed mechanism is unclear so far. Plastid CK2 is a major Ser/Thr-specific protein kinase in the chloroplast stroma. The activity of this kinase is regulated by redox signals. The expression of the gene encoding this enzyme is upregulated by abscisic acid (ABA) or heat treatment [51]. Mutants lacking this enzyme are more sensitive to heat treatment, and many genes that are upregulated in response to heat stress are instead inhibited. In addition, the transcription levels of several key genes of chloroplast retrograde signalling, including *PTM* (coding a chloroplast envelope-bound plant homeodomain (PHD) transcription factor with transmembrane domains), *ABI4* (*ABA insensitive 4*), and *RPS1*, are significantly reduced. Therefore, CK2 may play a role in plant stress resistance by regulating key genes in chloroplast retrograde signalling. 

Subsequently, intermediate signalling components between the plastid and nucleus are required to transmit the signalling from chloroplasts, in order to regulate plant resistance to heat stress. They can be Ca^2+^ ions, the HSP90 associated complex, and PTM, as well reviewed by Sun and Guo [42]. However, the plastid signal(s) in cytosol are still largely unknown. In the nucleus, some transcription factors, such as HSFA2, HY5, GLKs, and ABI4, were considered to respond to heat-induced chloroplast retrograde signalling. They can activate HSFs and other target genes that can improve plant heat tolerance [42].

## 4. Heat Tolerance of Plants with the Involvement of Chloroplast Proteins

After chloroplasts sense heat stress and subsequently transmit the heat signal to the nucleus, plants can activate many molecular chaperones and change their growth state by reprograming nucleus gene expression [52]. Among these newly expressed chaperones, some will be imported into chloroplasts to protect important proteins that may be easily destroyed by heat stress, thereby improving the heat tolerance of the plant to a certain extent. The most widely known chaperones are HSPs. HSPs are a group of heat-induced and evolutionarily conserved proteins that locate to the cytosol, chloroplast, mitochondria and nucleus, and other organelles. They are classified into a number of conserved protein families based on their molecular weight: HSP100s, HSP90s, HSP70s, HSP60s, HSP40s/DnaJ, and the small HSPs (sHSPs) [53,54]. As molecular chaperones, HSPs can prevent protein denaturation and aggregation, therefore improving heat tolerance of proteins [55]. For example, the sole chloroplast-targeted HSP100 protein in *Arabidopsis*, APG6/ClpB3, is necessary for plants to tolerate high temperatures and survive under heat stress [56]. It functions in reactivation of misfolded deoxyxylulose 5-phosphate synthase (DXS), the first enzyme of the plastidial isoprenoid pathway [57]. The chloroplast-targeted LeHSP100/ClpB in the tomato is believed to have the ability to protect PSII from heat stress damage [58]. In *Arabidopsis*, there are two stromal HSP70s, denoted cpHsc70-1 and cpHsc70-2. The transcripts of *cpHsc70-1* and *cpHsc70-2* are abundant in almost all tissues. It is demonstrated that cpHsc70-1 is necessary for plant development and is also very important for the heat tolerance of germinating seeds [59]. Overexpression of the chloroplast-localized HSP40/DnaJ chaperone LeCDJ1, functional in the protection of PSII, increases the heat tolerance of the tomato [60]. The other HSP40/DnaJ protein, LeCDJ2, as a co-molecular chaperone of cpHsc70, maintains Rubisco activity during heat stress [61]. Decreasing the expression of CaHSP60-6 in *Capsicum annuum* L. (pepper) increased the sensitivity of pepper to heat stress [62].

Plant sHSPs have a molecular size ranging from 16 to 42 kDa. They are classified into different subfamilies and localized to distinct subcellular compartments [63]. Most sHSPs are highly expressed under heat stress and protect proteins from denaturation under heat stress. In different plants, chloroplast sHSPs can bind to the thylakoid membrane and protect PSII, thereby helping the cell resist heat stress [64]. For example, *Arabidopsis* has as many as 19 sHSPs; only HSP21 is predicted to localize in the plastid [65]. HSP21 is a nuclear-encoded sHSP that is located in the chloroplast and plays an important role in the protection of PSII against heat stress [49]. Lack of HSP21 severely hinders seedling development. HSP21 can interact with the chloroplast nucleoid protein pTAC5 to maintain chloroplast-encoded RNA polymerase. Therefore, this protein is essential for chloroplast development [65]. HSP26.8a in the chloroplast of creeping bentgrass (*Agrostis stolonifera* L.) also responds to heat stress. In contrast, it is a negative regulator of heat stress [66]. The heat memory gene *FaHSP17.8-CII* of tall fescue (*Festuca arundinacea*) boosts heat tolerance in plants by altering PSII and ROS signalling [67]. In C4 and crassulacean acid metabolism plants, many chloroplast-localized sHSPs are the key components to improve the plant heat tolerance [68]. For example, chloroplast sHSP26 enhances the chloroplast expression of maize under heat stress by interacting with ATP synthase, chlorophyll *a*/*b*-binding proteins, and reaction centre proteins of PSI [69]. In green algae, *Chlamydomonas* HSP22E/F binds to unfolded chloroplast proteins under heat stress and forms a high-molecular-weight complex, thus preventing cytotoxicity due to protein accumulation [70].

In addition to HSPs, other proteins localized to chloroplasts that can respond to heat stress contribute to plant heat tolerance to varying degrees too. First, the plastid proteins that can suppress or scavenge ROS may contribute to plant heat tolerance. For example, chloroplast TRXL1 is reduced, is partly degraded, and alters its chloroplast localization under heat stress conditions [71]. TRXL1 positively regulates malate levels via activation of NADP-dependent malate dehydrogenase (NADP-MDH) to suppress ROS formation. Therefore, it has the ability to regulate heat tolerance and the disease resistance of plants. TRXL1 is degraded by the CLP protease complex and is protected by chaperonin CPN60A [71]. When *Arabidopsis* overexpressed ascorbate peroxidase (APX) from the heat-tolerant red algae *Cyanidioschyzon merolae*, it localized to the chloroplast stroma and significantly improved the ROS scavenging and heat resistance of *Arabidopsis* [72]. Tomato SlSNAT is a key enzyme that participates in the synthesis of melatonin, an effective ROS scavenger. SlSNAT is located in the chloroplast and interacts with HSP40 to enhance the heat tolerance of the tomato [73]. Overexpressing *SlSNAT* upregulated melatonin accumulation, ROS scavenging, Rubisco protection, and the expression of heat response genes in response to heat stress. Second, proteases that function in degrading destroyed chloroplast proteins is important to plant heat tolerance. FtsH11 is a protease in chloroplasts. Plants lacking FtsH11 showed a reduction in photosynthetic efficiency under high-temperature conditions, but their chlorophyll fluorescence parameters under optimal temperatures are not significantly different from those of the wild-type [74]. These results indicate that FtsH11 is a protein necessary for the maintenance of a sufficient photosynthesis rate under moderately high-temperature conditions, and it plays a key role in maintaining the thermal stability and integrity of the photosynthetic system [74]. Third, enzymes catalysing lipid metabolism in chloroplast are required for plant heat tolerance. GPAT is the first enzyme in the metabolism pathway of glycerolipid biosynthesis in chloroplasts [75]. Overexpressing sweet pepper (*Capsicum annuum* var. *grossum*) GPAT significantly increases the heat tolerance of the photosynthetic organs of tobacco [76].

## 5. Outlook

Chloroplasts play an important role in plant heat sensing, transmission of heat signals, and improvement in plant heat tolerance (Table 1). However, the contribution of chloroplasts to plant heat tolerance and the involved mechanisms are not well investigated. Using “(plant) and (heat)” as the searching key words in the PubMed website (https://pubmed.ncbi.nlm.nih.gov/, accessed on 22 August 2021), there were 26,332 results from 2000. While using “(chloroplast) and (heat)” as the key words to search, only 924 articles came out. One possible reason is that the chloroplast is too important to plants. Plants lacking important chloroplast proteins often show more easily observed phenotypes under non-stress conditions. Therefore, researchers did not focus on the proteins’ roles in the plant heat response or tolerance. Another possible reason is that the chloroplast retrograde signalling pathways are not well studied; their roles in chloroplast heat signal transmission have an insufficient research basis. Through continuous in-depth research, we believe that more molecular mechanisms underlying the chloroplast’s involvement in plant heat resistance will be elucidated, and more ways of improving the heat tolerance of plants through chloroplasts or chloroplast proteins will be discovered. Current research shows that increasing the abundance of proteins such as HSPs, TRXL1, SNAT, and GPAT in chloroplasts improves the heat tolerance of plants without causing significant defects in plant growth or development [58,71,73,76]. Future studies should also explore the detailed mechanism and the contributions of these or more genes to crop heat resistance, strive to find natural or induced mutants of these genes in crops, and find good heat-resistant materials for crop breeding. It is hoped that the breeding of excellent heat-resistant varieties can be accelerated to cope with the harm of global warming and extreme weather to crop production.

## Figures and Tables

**Table 1:** Summary of the responses of chloroplast and chloroplast retrograde signalling to heat, and chloroplast-related plant heat tolerance.

	Plant System	Related Physiological Processes	Proteins or Signal Molecules [Ref.]
Part I: Chloroplast are sensitive to heat stress:			
PSII	Plants	Photosynthesis	D1/D2 [12,13,14,15,16,17,18]
Protein translation system	*Arabidopsis*	Protein translation	EF-Tu [23,24,25], RPS1 [26]
Chlorophyll metabolism	Bentgrass *Arabidopsis*	Chlorophyll degradation Chlorophyll turnover	PPH [31] CHLG [32], CLD1 [33]
Dark reaction of photosynthesis	Plants	Photosynthesis	Rubisco and RCA [35,36,37]
Part II: Chloroplast retrograde signalling that responds to heat stress			
1. Calcium ion concentration	*Arabidopsis*	Calcium signalling	CAS [39]
2. ROS	Plants *Arabidopsis* Plants		Singlet oxygen [41] β-cyclocitral [43] H_2_O_2_ [44]
3. Metabolic molecules	*Chlamydomonas* *Arabidopsis*	Tetrapyrrole metabolism Tocopherol and sulfur metabolism	Mg-Proto IX and hemin [47] Vitamin E-PAP-miR398 [48]
4. Components in chloroplast	*Arabidopsis* *Arabidopsis* *Arabidopsis* *Arabidopsis*	Chlorophyll biosynthesis Protein translation Transcription and plastid translation Protein phosphorylation	GUN5 [49] RPS1 [26] CIA2 [50] CK2 [51]
5. Components out of chloroplast	*Arabidopsis* *Arabidopsis* *Arabidopsis*, tomato et al. *Arabidopsis*	Maintaining signalling proteins/Heat Transcription factor Transcription factor/Heat Transcription factor	HSP90 [42,76] PTM-ABI4 [42] HSFA2 [26,42] HY5, GLKs [42]
Part III: Chloroplast proteins contribute to plant heat tolerance			
1. HSPs a. HSP100s b. HSP70s c. HSP60s d. HSP40s e. sHSPs	*Arabidopsis*, Tomato *Arabidopsis* Pepper Tomato *Arabidopsis* Creeping bentgrass Tall fescue Maize *Chlamydomonas*	Protein quality control Protein quality control Protection of PSII, Rubisco Plastid transcription/PSII stabilization Thermomemory Protection of photosynthetic protein Protection of photosynthetic protein	APG/ClpB3 [56,57], LeHSP100 [58] cpHsc70-1 [59] CaHSP60-6 [62] LeCDJ1 [60], LeCDJ2 [61] HSP21 [65] HSP26.8a [66] FaHSP17.8-CII [67] sHSP26 [69] HSP22E/F [70]
2. ROS related	*Arabidopsis* *C. merolae* Tomato	Regulation of MDH ROS-scavenging Melatonin biosynthesis	TRXL1 [71] APX [72] SlSNAT [73]
3. Protease	*Arabidopsis*	Protein quality control	FtsH11 [74]
4. Lipid metabolism	Sweet pepper	Glycerolipid biosynthesis	GPAT [75]

## Abbreviations

Ca	calcium
ROS	reactive oxygen species
HSF	heat shock transcription factor
HSP	heat shock protein
PSI	photosystem I
PSII	photosystem II
Rubisco	Ribulose-1,5-bisphosphate carboxylase/oxygenase
LHCI	Light-harvesting complex I
EF-Tu	Translation elongation factor Tu
RPS1	Ribosomal protein S1
PPH	Pheophytinase
CHLG	Chlorophyll synthase
CLD1	Chlorophyll dephytylase1
CO_2_	carbon dioxide
RCA	Rubisco activase
CAS	Ca^2+^-sensing protein
H_2_O_2_	hydrogen peroxide
Mg-Proto IX	magnesium-protoporphyrin IX
PAP	3′-phosphoadenosine 5′-phosphate
GUN5	Genomes uncoupled 5
CIA2	Chloroplast import apparatus 2
CK2	Casein kinase 2
PTM	PHD type transcription factor with transmembrane domains
ABI4	ABA insensitive 4
HY5	Long hypocotyl 5
GLK	Golden 2-like
APG6	Albino or pale-green 6
Hsc70	Heat shock cognate protein 70
CDJ	Chloroplast DnaJ
pTAC5	Plastid transcriptionally active 5
TRXL1	Thioredoxin-like
MDH	Malate dehydrogenase
CPN60A	Chaperonin 60A
APX	Ascorbate peroxidase
SNAT	Serotonin N-acetyltransferase
FtsH	Filamentation temperature-sensitive H
GPAT	Glycerol-3-phosphate acyltransferase
